# Randomized controlled trial data for successful new drug application for rare diseases in the United States

**DOI:** 10.1186/s13023-023-02702-9

**Published:** 2023-04-19

**Authors:** Yosuke Kubota, Mamoru Narukawa

**Affiliations:** 1 Department of Clinical Medicine (Pharmaceutical Medicine), Graduate School of Pharmaceutical Sciences, Kitasato University, 5-9-1 Shirokane, Minato-ku, Tokyo, 108-8641 Japan; 2 Development, Astellas Pharma Inc, Tokyo, 103-8411 Japan

**Keywords:** Rare diseases, Orphan drugs, Clinical trials, Randomization, Efficacy endpoint, Logistic regression analysis

## Abstract

**Background:**

Randomized controlled trial (RCT) data have important implications in drug development. However, the feasibility and cost of conducting RCTs lower the motivation for drug development, especially for rare diseases. We investigated the potential factors associated with the need for RCTs in the clinical data package for new drug applications for rare diseases in the United States (US). This study focused on 233 drugs with orphan drug designations approved in the US between April 2001 and March 2021. Univariable and multivariable logistic regression analyses were conducted to investigate the association between the presence or absence of RCTs in the clinical data package for new drug applications.

**Results:**

Multivariable logistic regression analysis showed that the severity of the disease outcome (odds ratio [OR] 5.63, 95% confidence interval [CI] 2.64–12.00), type of drug usage (odds ratio [OR] 2.95, 95% confidence interval [CI] 1.80–18.57), and type of primary endpoint (OR 5.57, 95% CI 2.57–12.06) were associated with the presence or absence of RCTs.

**Conclusions:**

Our results indicated that the presence or absence of RCT data in the clinical data package for successful new drug application in the US was associated with three factors: severity of disease outcome, type of drug usage, and type of primary endpoint. These results highlight the importance of selecting target diseases and potential efficacy variables to optimize orphan drug development.

**Supplementary Information:**

The online version contains supplementary material available at 10.1186/s13023-023-02702-9.

## Introduction

Because the market for drugs for rare diseases (orphan drugs) is fragmented, there is less competition among new drugs. Generic drugs are less likely to invade the market, even after the patent expires. Thus, it is possible to monopolize the market for a long time while maintaining a higher price [1]. However, orphan drug development is associated with a higher risk due to the lack of sufficient information on the disease course and difficulty in conducting clinical trials due to the small number of patients. For this reason, major pharmaceutical companies have a history of restraining investment in orphan drugs as a high-risk, high-return niche. In recent years, however, the development of new drugs in areas with a large number of patients has almost become saturated. Competition has intensified, inspiring major pharmaceutical and emerging biotech companies to maintain and improve their business by developing orphan drugs [2].

In the United States (US), rare diseases are defined as “diseases with fewer than 200,000 patients in the US” [3]. It is estimated that there are approximately 7,000 rare diseases worldwide, and approximately 30 million people in the US are afflicted with a rare disease [4, 5]. The US government has supported the development of orphan drugs since 1983 [6]. Currently, the exclusivity period is set at seven years, 50% of the costs are tax-deductible, and a new drug application fee to the Food and Drug Administration (FDA) is exempt [7].

Despite these efforts, the high cost of clinical trials, difficulty in patient recruitment, and strict regulatory requirements for new drug reviews have been highlighted as hurdles to developing orphan drugs. In many cases, patients are scattered across the country or region, specialized medical institutions are not always located nearby, and accurate diagnosis takes time, which makes conducting clinical trials difficult. Although consideration is given to such circumstances, guidance by regulatory authorities recommends generating as much high-quality evidence as possible [8–10].

Pharmaceutical companies and regulatory authorities are constructing an optimal orphan drug development strategy that considers the required quality and quantity of evidence. The feasibility of clinical trials for rare diseases is also discussed on a case-by-case basis. Incorporating information outside clinical trials, such as natural history study data, research reports, and the use of real world data, to generate comprehensive evidence is also drawing attention due to the various biases considered [11–14]. It has been reported that clinical trials for rare diseases tended to have fewer blinded and randomized trials than those for non-rare diseases [15, 16].

A randomized clinical trial (RCT) is still one of the most important elements in discussing high-quality evidence [17–19]. Conventional RCTs with a comparator and proper sample size for statistical analyses are the scientific standard [20]. However, the feasibility of conducting clinical trials and an increase in associated costs can also lower the motivation for development. The efficient implementation of RCTs is also being investigated by incorporating methods such as adaptive designs, crossover trials, and early escape designs according to the characteristics of targeted rare diseases [21–26]. As described above, the utilization of RCT data is considered to have important implications for drug development in rare diseases.

In a previous study, we investigated the presence or absence of RCTs in the clinical data package for new drug applications in Japan and reported that the prevalence of the disease and type of primary endpoint was associated with the necessity of an RCT [27]. However, there is room for further research on the factors associated with the necessity and the feasibility of RCT in application packages. Considering the timing of drug development and approval among countries or regions and differences in the drug development strategy, we conducted the present study on orphan drugs in the US, where more active drug development is being carried out.

## Methods

### Data source and items

Newly approved drugs with an orphan drug designation (ODD) in the US between April 1, 2001, and March 31, 2021 were extracted from the approved drug list on the website of the US FDA (https://www.accessdata.fda.gov/scripts/opdlisting/oopd/). Among them, drugs with missing patient prevalence data, drugs for diagnosis or surgery aid, and drugs that were approved without clinical trial data were excluded from the analysis.

Furthermore, for each approved drug, a pivotal trial, typically a clinical trial to confirm the safety and efficacy of the drug, was selected from the FDA review report. This selection was performed based on the following hierarchy: trial phase (phase 3, 2), trial design (RCT, single-arm trial [SAT]), and the number of enrolled patients (large, small). The pivotal trial design was categorized as RCT or SAT. If several indications were approved in a single new drug application (NDA) based on different pivotal trials, each indication was treated as distinct approval. The following data were extracted for the selected drugs: approval year, prevalence of the target disease, modality, Anatomical Therapeutic Chemical (ATC) classification [28], target age segment, type of primary endpoint in the selected pivotal trial, severity of the disease outcome, prior approval outside the US, availability of alternative treatment(s) in the US, type of drug usage (mono or combination therapy), and designations of special regulatory pathways in the US (fast track, breakthrough therapy, priority review, and accelerated approvals).

When assessing the potential association with the presence or absence of an RCT in the clinical data package as a response variable, the following factors were considered as explanatory variables: prevalence (< 1/100,000 vs. ≥ 1 /100,000 patients), severity of disease outcome (high mortality vs. others), type of drug usage (monotherapy vs. combination therapy), therapeutic classification (oncology vs. non-oncology), target age segment (children with or without adult vs. adult only), and type of primary endpoint (biomarker vs. clinical outcome).

The severity of the disease outcome was categorized as high mortality or others (e.g., progressing disability early in life or chronic but manageable with treatments and lifestyle adjustments) based on the information available on the review reports and the Orphanet website (https://www.orpha.net/consor/cgi-bin/index.php). Subsequently, some of the high-mortality diseases were changed to others, based on whether the mortality was manageable with alternative treatments, to reflect the impact of the available alternative treatments on the severity of disease outcome in the US.

The primary endpoint of the selected pivotal trial was categorized as a biomarker or clinical outcome. We referred to the pharmacodynamic or response biomarker definition, “a biomarker used to show that a biological response has occurred in an individual who has been exposed to a medical product or an environmental agent.” Clinical outcome was defined as “an outcome that describes or reflects how a patient feels, functions, or survives [29].” In case of multiple primary endpoints, clinical outcomes were preferentially selected.

We assessed these two explanatory variables independently. Disagreements between the reviewers were resolved by consensus, and a 100% consensus was reached for all data included in the analysis.

The definitions and criteria of the other explanatory variables are summarized in **Online Resource 1** (Supplementary Information).

### Statistical analysis

The associations between the response variable and each of the explanatory variables were assessed using univariable and multivariable logistic regression analyses to calculate the crude odds ratio (OR) and 95% confidence interval (CI). In the multivariable logistic regression analysis, we removed therapeutic classification (oncology vs. non-oncology) from the explanatory variables because it showed a high association with the severity of the disease outcome (Cramer’s V > 0.5). Other explanatory variables did not show a strong association with each other and were included in the multivariable logistic regression analysis. Statistical significance was set at *P* < 0.05. StatsDirect version 3.3.5 software was used for all the statistical analyses.

## Results

**Fig. 1:**
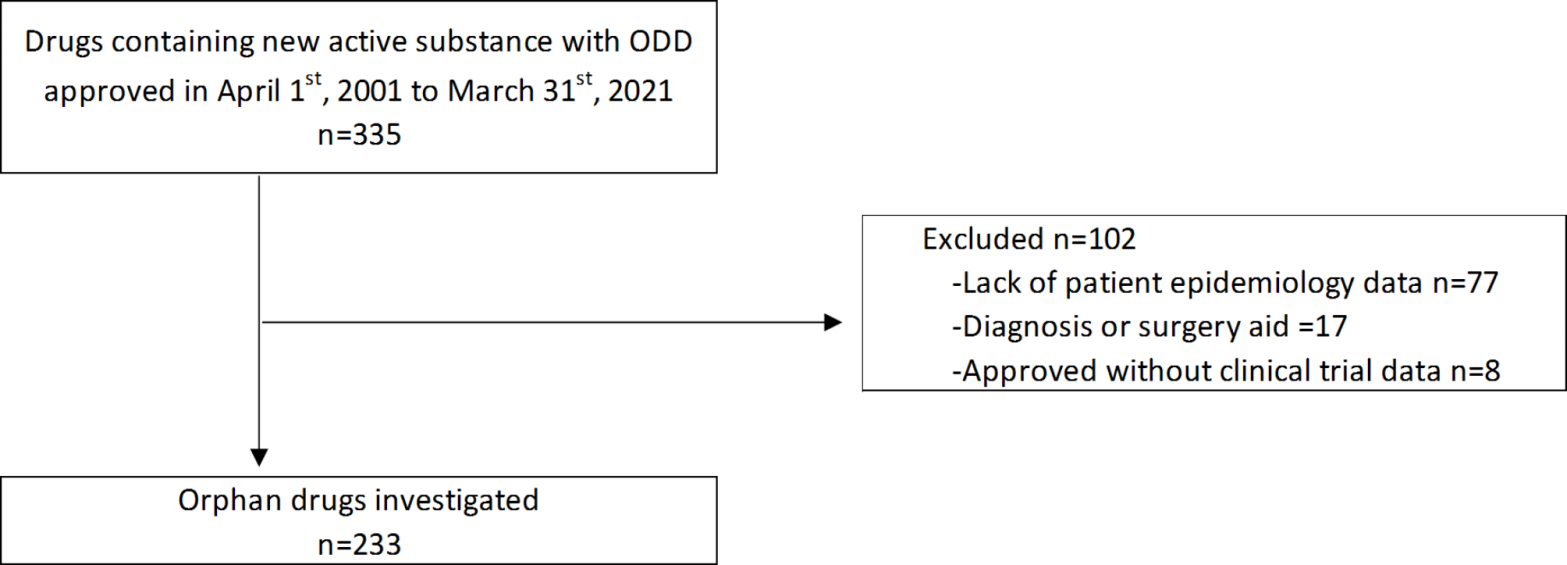
Flow diagram of drug selection

A total of 335 drugs with ODD containing new active substances approved in the US between April 1, 2001, and March 31, 2021 were identified. Among these, 233 drugs with ODD were selected for the analysis (Fig. 1).

The characteristics of the selected orphan drugs are summarized along with the design of the pivotal trial, RCT, or SAT (Table 1). Approximately two-thirds of orphan drugs (151 of 233) had a pivotal RCT, and one-third (82 of 233) had a pivotal SAT in the clinical data package. Most drugs (190 of 233) were first approved worldwide. 81% of orphan drugs in SAT targeted high mortality diseases, while 44.4% of the drugs in an RCT targeted high mortality diseases.

**Table 1:** Characteristics of orphan drugs

	Single-arm trial (n=82)	Randomized controlled trial (n=151)		Total (n=233)
**Approval year**				
2001–2005	5 (6.1%)		17 (11.3%)	22
2006–2010	9 (11.0%)		18 (11.9%)	27
2011–2015	28(34.1%)		42 (27.8%)	70
2016–2021	40 (48.8%)		74 (49.0%)	114
**Prevalence**				
1–5 / 10,000	38 (46.3%)		66 (43.7%)	104
1–9 / 100,000	31 (37.8%)		59 (39.1%)	90
1–9 / 1,000,000	6 (7.3%)		21 (13.9%)	27
< 1 / 1,000,000	7 (8.5%)		5 (3.3%)	12
**Modality**				
Chemical	52 (63.4%)		92 (60.9%)	144
Biologics	30 (36.6%)		59 (39.1%)	89
**ATC code**				
A (Alimentary tract and metabolism)	6 (7.3%)		20 (13.2%)	26
B (Blood and blood forming organs)	9 (11.0%)		20 (13.2%)	29
C (Cardiovascular system)	1 (1.2%)		8 (5.3%)	9
D (Dermatologicals)	0		2 (1.3%)	2
G (Genito urinary system and sex hormones)	1 (1.2%)		0	1
H (Systematic hormonal preparations, excl. sex hormones and insulins)	0		7 (4.6%)	7
J (Antiinfectives for systemic use)	0		3 (1.3%)	3
L (Antineoplastic and immunomodulating agents)	64 (78.0%)		52 (34.4%)	116
M (Musculo-skeletal system)	1 (1.2%)		8 (5.3%)	9
N (Nervous system)	0		17 (11.3%)	17
P (Antiparasitic products, insecticides and repellents)	0		5 (3.3%)	5
R (Respiratory system)	0		4 (2.6%)	4
S (Sensory organs)	0		3 (2.0%)	3
V (Various)	0		2 (1.3%)	2
**Target age segment**				
Children with or without adult	24 (29.3%)		55 (36.4%)	79
Adult only	58 (70.7%)		96 (63.6%)	154
**Primary efficacy endpoint**				
Pharmacodynamic/Response biomarker	69 (84.1%)		67 (44.4%)	136
Clinical outcome	13 (15.9%)		84 (55.6%)	97
**Severity of the disease outcome**				
High mortality	67 (81.7%)		67 (44.4%)	134
Others	15 (18.3%)		84 (55.6%)	99
**Prior approval outside of US**				
Yes	9 (11.0%)		34 (22.5%)	43
No	73 (89.0%)		117 (77.5%)	190
**Alternative treatment in US**				
Not available	25 (30.5%)		51 (33.8%)	76
Exists	57 (69.5%)		100 (66.2%)	157
**Drug usage**				
Mono therapy	78 (95.1%)		128 (84.8%)	206
Combination therapy	4 (4.9%)		23 (15.2%)	27
**Designation**				
Fast track				
Yes	17 (20.7%)		51 (33.8%)	68
No	65 (79.3%)		100 (66.2%)	165
Breakthrough therapy				
Yes	31 (37.8%)		38 (25.2%)	69
No	51 (62.2%)		113 (74.8%)	164
Priority reivew				
Yes	52 (63.4%)		87 (57.6%)	139
No	30 (36.6%)		64 (42.4%)	94
Accelerated approval				
Yes	43 (52.4%)		31 (20.5%)	74
No	39 (47.6%)		120 (79.5%)	159

Table 2 shows the results of the univariable and multivariable analyses to investigate the associations between the presence or absence of RCT data in the clinical data package and explanatory variables. Univariable analyses showed that the following factors were associated with the presence or absence of RCT data in the clinical data package for NDA in the US: (1) severity of disease outcome, (2) type of drug usage, and (3) type of primary endpoint. The results of the multivariable analysis also suggested that severity of disease outcome (OR 5.63, 95% CI 2.64–12.00), type of drug usage (OR 2.95, 95% CI 1.80–18.57), and type of primary endpoint (OR 5.57, 95% CI 2.57–12.06) were associated with the presence or absence of RCT data.

**Table 2:** Results of univariable and multivariable logistic regression analyses

Factor	Single-arm trial (n = 82)	Randomized controlled trial (n = 151)	Total (n = 233)	Univariable logistic regression analysis			Multivariable logistic regression analysiss		
				Odds ratio	95% CI	*p* value	Odds ratio	95% CI	*p* value
**Prevalence**									
< 1/100,000	13 (15.9%)	26 (17.2%)	39	Reference			Reference		
≥ 1/100,000	69 (84.1%)	125 (82.8%)	194	0.91	0.44–1.88	0.7899	1.31	0.52–3.34	0.561
**Severity of the disease outcome**									
High mortality	67 (81.7%)	67 (44.4%)	134	Reference			Reference		
Others	15 (18.3%)	84 (55.6%)	99	5.60	2.94–10.68	< 0.0001*	5.63	2.64-12.00	< 0.0001*
**Drug usage**									
Mono therapy	78 (95.1%)	128 (84.8%)	206	Reference			Reference		
Combination therapy	4 (4.9%)	23 (15.2%)	27	3.50	1.17–10.51	0.0253*	2.95	1.80-18.57	0.0032*
**Target age segment**									
Children with or without adult	24 (29.3%)	55 (36.4%)	79	Reference			Reference		
Adult only	58 (70.7%)	96 (63.6%)	154	0.72	0.40–1.29	0.2714	1.72	0.75–3.97	0.2027
**Primary efficacy endpoint**									
Pharmacodynamic/Response biomarker	69 (84.1%)	67 (44.4%)	136	Reference			Reference		
Clinical outcome	13 (15.9%)	84 (55.6%)	97	6.65	3.39–13.06	< 0.0001*	5.57	2.57–12.06	0.0001*
**Therapeutic clasiffication**									
Oncology	63 (76.8%)	46 (30.5%)	109	Reference					
Nononcology	19 (23.2%)	105 (69.5%)	124	7.57	4.08–14.06	< 0.0001*		N/A	

*P value < 0.05

## Discussion

In this study, we investigated the presence or absence of RCTs in the clinical data package of new drug applications with ODD in the US. We found that the severity of disease outcome, type of drug usage, and type of efficacy endpoint were associated with the presence or absence of RCTs. These results suggest the importance of understanding the target disease and its treatment environment.

Disease severity, defined by high mortality, was positively associated with the absence of RCT in the package, implying that RCTs are more difficult to conduct in such disease areas. This result was expected because there is no authorized treatment available for most rare diseases [30], and it has been pointed out that it is ethically difficult to conduct a comparative study with a placebo group when there is no standard of care [31]. In such cases, conditional approval based on the totality of evidence with SAT data and other external data under the accelerated approval system may be granted in the US. Although it is necessary to reinforce the evidence through confirmatory trials after conditional approval, early access to treatment options is favorable for the patients suffering from these serious diseases.

Second, the utilization of combination therapy for a test arm showed a positive association with the presence of RCT. From an ethical perspective, this is another expected result. When the test arm is a combination therapy in which an investigational drug is added to a standard therapy, a standard of care is warranted, which would lower ethical barriers and make it easier to justify conducting RCTs to pursue high-level evidence.

Third, the use of clinical outcomes as an efficacy endpoint was positively associated with the presence of an RCT. This result is similar to that of our previous study using the Japanese data package [27]. In general, biomarkers can be utilized as efficacy endpoints when an appropriate understanding of the disease has been achieved, and their correlation with the true endpoint has been appropriately justified. Thus, biomarkers based on laboratory test values and radiographic imaging make it possible to obtain early clinical results with a small sample size and lower the possibility of introducing bias [29]. Based on these considerations, it would be natural for clinical trials with smaller sample sizes to be preferred for rare diseases, as it is difficult to secure enough patients to participate in the trials [15, 21]. The trend of higher utilization of SAT in the clinical data package of orphan drugs compared to those targeting non-rare diseases is consistent with the results of a previous study [15]. The findings of the present study and our previous study in Japan [27] suggest the importance of this factor in considering the necessity and feasibility of RCTs.

It was unexpected for no association to be found between the prevalence of the disease and the presence or absence of RCT. In general, an appropriate sample size is required to conduct an RCT. Thus, we assumed that a lower prevalence of the target disease would be associated with fewer RCTs due to decreased feasibility. In our previous study in Japan, a negative association was confirmed between RCTs and lower prevalence disease prevalence [27]. Although this inconsistency may be due to differences in the drugs targeted in each study, and the sources of prevalence may also be attributable, the established multi-regional clinical trial (MRCT) approach in the US may have contributed to enhancing the feasibility of RCTs. The US has an approximately 2.5 times larger population compared to Japan. Thus, the absolute number of target patients would be relatively larger in the US and further increases when MRCT countries are included. As a result, it is speculated that expectations of evidence generation via RCTs are relatively unconstrained by prevalence, even in rare diseases in the US. This result is consistent with previous reports that showed a similar proportion of RCTs over diseases with various prevalence rates [32, 33].

This study had several limitations. First, the study was conducted only for approved drugs, which could be regarded as a source of bias. The information available on negative results is less extensive than that available for approved drugs. Second, the data were collected only for drugs that had an ODD. There may be drugs that did not apply for ODD, although they met the ODD criteria. Third, the association between clinical equipoise or natural history data and the presence or absence of RCTs was not investigated. Clinical equipoise is an important ethical consideration for determining the feasibility of RCTs [34], and natural history data are another crucial factor to support the discussion on RCT or SAT from the perspective of totality of evidence in the clinical data package. However, it was difficult to quantify the equipoise and contribution of natural history data to various areas of drugs in this study.

Orphan drugs target various rare diseases with unmet medical needs. If drug development is conducted using SAT data based on sufficient research, discussion, and consideration, it will streamline the development timeline. Thus, patients with rare diseases and unmet medical needs may be afforded early access to these drugs. Conversely, RCTs may impact the feasibility and development period of drug development. However, they are recommended by various guidelines as the results obtained through RCTs are highly reliable and ultimately benefit patients. This argument is unavoidable in orphan drug development, emphasizing the importance of utilizing available evidence.

Our results indicated the importance of selecting a target disease and potential efficacy variables to be used in the clinical trials to examine the need for RCT data for NDA. From a pharmaceutical company perspective, target disease selection is one of the initial steps in drug development. Preparation of a clinical development plan including pivotal study designs is initiated during this step, which significantly impacts the costs and timelines of drug development, consequently affecting corporate investment decisions. In addition, we believe that strategic collection of relevant information is necessary to establish efficacy variables for potential drug development. This can be achieved through collaboration among the industry, government, and academia, which will help facilitate orphan drug development.

## Conclusions

Our results indicated that the presence or absence of RCT data in the clinical data package for NDA in the US was associated with three factors: severity of disease outcome, type of drug usage, and type of primary endpoint. Due to the small population of rare diseases, discussion on the necessity of RCT data tends to focus on the feasibility of conducting the clinical trials. While it is an unavoidable nature of orphan drug development, our results indicated the importance of selecting a target disease and potential efficacy variables to be used in the clinical trials to examine the need for RCT data for NDA. We hope that the results of this study will contribute to improving the predictability of the need for RCT data, optimizing investment in new drug development, and enhancing strategic collection of relevant information to establish efficacy variables for potential orphan drug development.

## Electronic supplementary material

Below is the link to the electronic supplementary material.

Supplementary Material 1

## Data Availability

Data will be made available on request.
