# Supplementary material for: Randomized controlled trial data for successful new drug application for rare diseases in the United States
Source: Orphanet J Rare Dis. 2023 Apr 19;18:89. doi: 10.1186/s13023-023-02702-9 (PMC10114466; doi:10.1186/s13023-023-02702-9)
Supplement: Supplementary file 1 — Supplementary Material 1 [file 13023_2023_2702_MOESM1_ESM.docx]

**Online Resource 1**

**Randomized controlled trial data for new drug application for rare diseases in the United States**

Yosuke Kubota ^1,2*^・Mamoru Narukawa^2^

^1^ Department of Clinical Medicine (Pharmaceutical Medicine), Graduate School of Pharmaceutical Sciences, Kitasato University, 5-9-1 Shirokane, Minato-ku, Tokyo 108-8641, Japan

^2^ Development, Astellas Pharma Inc., Tokyo 103-8411, Japan

*Corresponding author: Department of Clinical Medicine (Pharmaceutical Medicine), Graduate School of Pharmaceutical Sciences, Kitasato University, 5-9-1 Shirokane, Minato-ku, Tokyo 108-8641, Japan; dl20402@st.kitasato-u.ac.jp

**1.** Prevalence (<1/100,000 vs. ≥1/100,000)

The prevalence of patient-targeted drugs was obtained from Orphanet (<https://www.orpha.net/consor/cgi-bin/index.php>) and the Surveillance, Epidemiology, and End Results (https://seer.cancer.gov/). Additionally, the number of patients for target drugs was obtained from review reports and divided by the United States national population (3.32 million) in 2022 to calculate the prevalence.

The largest estimated prevalence among these sources was selected in case of conflict. Prevalence was categorized into < 1/1,000,000, < 1–9/1,000,000, < 1–9/100,000, and < 1–5/10,000 based on the Orphanet range (http://www.orphadata.org/cgi-bin/epidemio.html). For statistical analysis, these categories were categorized as < 1/100,000 versus ≥ 1/100,000.

**2.** Therapeutic area (Oncology vs Non-oncology)

The approved medicines were categorized into *oncology* or *non-oncology* based on ATC codes.

**3.** Patient age segment (Children with or without adult vs. adult only)

Diseases were categorized as *Adults and Children* (diseases affecting adults and children or children only) and *Adult only* (diseases affecting adults only). If in the package insert, under *Indications*, *Dosage and Administration,* or *Pediatric Use*, there is an indication of their applicability to children, the drug is categorized under *Adult and Children.*
